# Antigenic Drift of the Pandemic 2009 A(H1N1) Influenza Virus in a Ferret Model

**DOI:** 10.1371/journal.ppat.1003354

**Published:** 2013-05-09

**Authors:** Teagan Guarnaccia, Louise A. Carolan, Sebastian Maurer-Stroh, Raphael T. C. Lee, Emma Job, Patrick C. Reading, Stephen Petrie, James M. McCaw, Jodie McVernon, Aeron C. Hurt, Anne Kelso, Jennifer Mosse, Ian G. Barr, Karen L. Laurie

**Affiliations:** 1 WHO Collaborating Centre for Reference and Research on Influenza, Victorian Infectious Diseases Reference Laboratory, Melbourne, Victoria, Australia; 2 Monash University, School of Applied Sciences, Churchill, Victoria, Australia; 3 Bioinformatics Institute (BII), Agency for Science, Technology and Research (A*STAR), Singapore; 4 National Public Health Laboratory, Communicable Diseases Division Ministry of Health, Singapore; 5 School of Biological Sciences (SBS), Nanyang Technological University (NTU), Singapore; 6 The University of Melbourne, Department Microbiology & Immunology, Melbourne, Victoria, Australia; 7 The University of Melbourne, Melbourne School of Population Health, Melbourne, Victoria, Australia; 8 Royal Children's Hospital, Murdoch Childrens Research Institute, Vaccine and Immunisation Research Group, Melbourne, Victoria, Australia; National Institutes of Health, United States of America

## Abstract

Surveillance data indicate that most circulating A(H1N1)pdm09 influenza viruses have remained antigenically similar since they emerged in humans in 2009. However, antigenic drift is likely to occur in the future in response to increasing population immunity induced by infection or vaccination. In this study, sequential passaging of A(H1N1)pdm09 virus by contact transmission through two independent series of suboptimally vaccinated ferrets resulted in selection of variant viruses with an amino acid substitution (N156K, H1 numbering without signal peptide; N159K, H3 numbering without signal peptide; N173K, H1 numbering from first methionine) in a known antigenic site of the viral HA. The N156K HA variant replicated and transmitted efficiently between naïve ferrets and outgrew wildtype virus *in vivo* in ferrets in the presence and absence of immune pressure. *In vitro*, in a range of cell culture systems, the N156K variant rapidly adapted, acquiring additional mutations in the viral HA that also potentially affected antigenic properties. The N156K escape mutant was antigenically distinct from wildtype virus as shown by binding of HA-specific antibodies. Glycan binding assays demonstrated the N156K escape mutant had altered receptor binding preferences compared to wildtype virus, which was supported by computational modeling predictions. The N156K substitution, and culture adaptations, have been detected in human A(H1N1)pdm09 viruses with N156K preferentially reported in sequences from original clinical samples rather than cultured isolates. This study demonstrates the ability of the A(H1N1)pdm09 virus to undergo rapid antigenic change to evade a low level vaccine response, while remaining fit in a ferret transmission model of immunization and infection. Furthermore, the potential changes in receptor binding properties that accompany antigenic changes highlight the importance of routine characterization of clinical samples in human A(H1N1)pdm09 influenza surveillance.

## Author Summary

Infection with influenza virus leads to significant morbidity and mortality. Annual vaccination may prevent subsequent disease by inducing neutralizing antibodies to currently circulating strains in the human population. To escape this antibody response, influenza A viruses undergo continuous genetic variation as they replicate, enabling viruses with advantageous antigenic mutations to spread and cause disease in naïve or previously immune or vaccinated individuals. To date, the 2009 pandemic virus (A(H1N1)pdm09) has not undergone significant antigenic drift, with the result that the vaccine remains well-matched and should provide good protection to A(H1N1)pdm09 circulating viruses. In this study, we induced antigenic drift in an A(H1N1)pdm09 virus in the ferret model. A single amino acid mutation emerged in the dominant surface glycoprotein, hemagglutinin, which had a multifaceted effect, altering both antigenicity and virus receptor specificity. The mutant virus could not be isolated using routine cell culture methods without the virus acquiring additional amino acid changes, yet was fit *in vivo*. The implications for surveillance of circulating influenza virus are significant as current assays commonly used to assess vaccine mismatch, as well as to produce isolates for vaccine manufacture, are biased against identification of viruses containing only this mutation.

## Introduction

The first influenza pandemic of the 21st century began in March 2009 with the emergence of a new swine-origin virus (A(H1N1)pdm09) which replaced the previous seasonal A(H1N1) [1]. Surveillance of circulating A(H1N1)pdm09 viruses has revealed some genetic variation in the hemagglutinin (HA) and neuraminidase (NA), but no significant antigenic changes have occurred over the past four years [2]. Moreover, the most recent circulating A(H1N1)pdm09 viruses remain antigenically closely related to the 2009 vaccine strain, A/California/7/2009 [3]. Serological studies estimated that during the first wave of infection in 2009, 34–43% of school-aged children and about 10% of adults were infected by A(H1N1)pdm09 viruses [4]. This level of natural exposure, combined with extensive vaccination and the ongoing circulation of these viruses since 2009, has established widespread immunity against the A(H1N1)pdm09 virus in the human population.

Influenza A viruses undergo continuous antigenic variation, ensuring efficient replication in the human host in the face of previous vaccination and/or infection (reviewed in [5]). Antigenic drift involves amino acid changes in antigenic regions of influenza proteins, facilitating escape from existing immunity. Drift mutations most commonly occur in the gene encoding the HA surface glycoprotein, which is the major target of neutralizing antibodies elicited as a result of vaccination and/or natural infection. Mutations are typically localized to antigenic sites surrounding the receptor binding site on the globular head of HA ([6]–[10], reviewed in [11]), consistent with the critical role of HA in binding to cell-surface receptors to initiate infection of respiratory epithelial cells.

A limited number of *in vivo* and *in vitro* studies have attempted to select influenza virus mutants in the presence of neutralizing antibodies [12]–[16]. A(H1N1), A(H3N8) and A(H3N2) viruses have been passaged multiple times through immunized mice [13] and once through dogs [14] and guinea pigs [17], respectively. A(H1N1)pdm09 virus has been cultured in embryonated hen's eggs in the presence of mouse monoclonal antibodies [16] or in MDCK cells in the presence of a human monoclonal antibody [15]. Resulting ‘immune escape mutants’ often express mutations that are closely linked with changes in HA receptor binding specificity and avidity for cell surface receptors [13], [16]. Immune pressure has also been shown to affect viral diversity [14], [18]. The selection of immune escape mutants in the presence of neutralizing antibodies has been proposed as a major factor driving evolution of HA in human influenza viruses. Early models proposed that passage of virus through individuals with different antibody specificities may induce sequential changes in antigenic regions, resulting in antigenic drift [19], [20]. More recently it has been postulated that alteration in the HA binding avidity for cell surface receptors drives antigenic drift and can occur independently, or, alongside variation in antigenicity as virus is passaged alternately through partially immune and naïve individuals [13].

Epithelial cells lining the airways of humans and ferrets express a similar pattern of sialylated receptors, enabling human influenza viruses to infect ferrets directly [21]. Following influenza infection, ferrets display similar disease symptoms and pathology to those observed in humans [22], [23]. Multiple immunizations with human influenza vaccines and adjuvant can protect ferrets from subsequent upper respiratory tract challenge with influenza virus [24]. In the absence of adjuvant, immunization with human influenza vaccine does not result in sterilizing immunity but significantly reduces viral load in the lower respiratory tract [25]. Serum from influenza-infected ferrets is commonly used in surveillance to assess antigenicity of influenza viruses circulating in the human population [26]. In this study, an A(H1N1)pdm09 virus A/Tasmania/2004/2009 was passaged by contact transmission in ferrets immunized with human A(H1N1)pdm09 vaccine and adjuvant. An antigenic escape mutant with altered receptor binding specificity emerged in two independent experiments. This escape mutant, which has also been detected in human surveillance studies, was difficult to detect and isolate using conventional *in vitro* methods.

## Results

### A(H1N1)pdm09 virus can transmit between ferrets in the presence of specific antibodies

A model of influenza transmission under immune pressure was established by suboptimal immunization of ferrets with the human monovalent A(H1N1)pdm09 influenza vaccine with adjuvant (MIV+IFA) or vaccine alone (MIV). A single immunization of human influenza vaccine with adjuvant does not induce sterilizing immunity in ferrets ([24] and data not shown). All animals immunized once with MIV+IFA had serum antibody titres ≥40 (geometric mean titre [GMT] 332) (measured by hemagglutination inhibition, HI), while only 31% of animals immunized twice with MIV had HI titres ≥40 (GMT 10) (**Table 1**). In preliminary experiments, we established that the A(H1N1)pdm09 virus A/Tasmania/2004/2009 was antigenically indistinguishable from the vaccine strain A/California/7/2009 by HI assay and grew to high titres in both the upper and lower respiratory tract of naïve ferrets (data not shown). Although gene sequencing detected five amino acid differences between the HA of A/Tasmania/2004/2009 (GISAID EPI_ISL_129743) and A/California/7/2009 (GenBank: CY058519) (N38N/S, P83S, S203T, T209K, I321V, **Table 2**), none were in the major antigenic regions. Amino acids in HA are referenced using H1 numbering without the signal peptide [27] in this manuscript.

**Table 1:** Serological responses following vaccination and infection.

A/Tasmania/2004/2009	Pre-contact transmission^a^		Post-contact transmission^b^	
HI titres	% HI titre ≥40	GMT (95% CI)	% HI titre ≥40	GMT (95% CI)
Naïve (n = 16)	0	5	56	73 (19, 280)
MIV (n = 16)	31	10 (6, 19)	62.5	84 (27, 263)
PBS+IFA (n = 16)	0	5	69	80 (25, 257)
MIV+IFA (n = 20)	100	332 (184, 597)	100	1114 (405, 3068)

a 3–13 weeks following last immunization.

b at least 3 days after detection of influenza virus in nasal wash by real time RT-PCR.

**Table 2:** Full genome sequencing of inoculum and influenza virus from peak day nasal wash of all R7 ferrets.

Comparison to MIV	HA	NA	M	NP	NS	PB1	PB2	PA
A/Tasmania/2004/2009 inoculum^a^	N38N/S^b^, P83S^b^, S203T^b^, T209K^b^, I321V^b^	V106I^b^, N248D^b^, S388T^b^		V100I^b^	I123V^b^		K312R^b^, N556S^b^	P224S^b^
NAÏVE A R7	G131S, L191I, R223Q						D87N	
NAÏVE B R7	D187E, L191I, R223Q						M66I	
MIV A R7	D187V, L191I, R223Q	M15I					D87N	
MIV B R7	L191I, R223Q					A643V, K691N	R293M	
PBS+IFA A R7	L191I, R223Q		P69P/A				R251K	R254R/Q
PBS+IFA B R7	L191I, R223Q		H214Y				D60N	
MIV+IFA A R7	D14E, N156K, L191I, R223Q		D21G (M2)	S3F^c^			N348S, M410V	
MIV+IFA B R7	N156K, L191I, R223Q	I46F						
N156K Naïve A R7	D14E, N156K, L191I, R223Q		D21G (M2), I28T (M2)	S3F^c^, M239V			N348S, M410V	
N156K Naïve B R7	K142N, N156K, L191I, R223Q	I46F						

a Differences between inoculum and vaccine strain, A/California/7/2009, are indicated.

b These mutations persisted in all ferrets, and are removed from R7 samples for clarity of Table.

c Nuclear localization signal.

**Figure 1:**
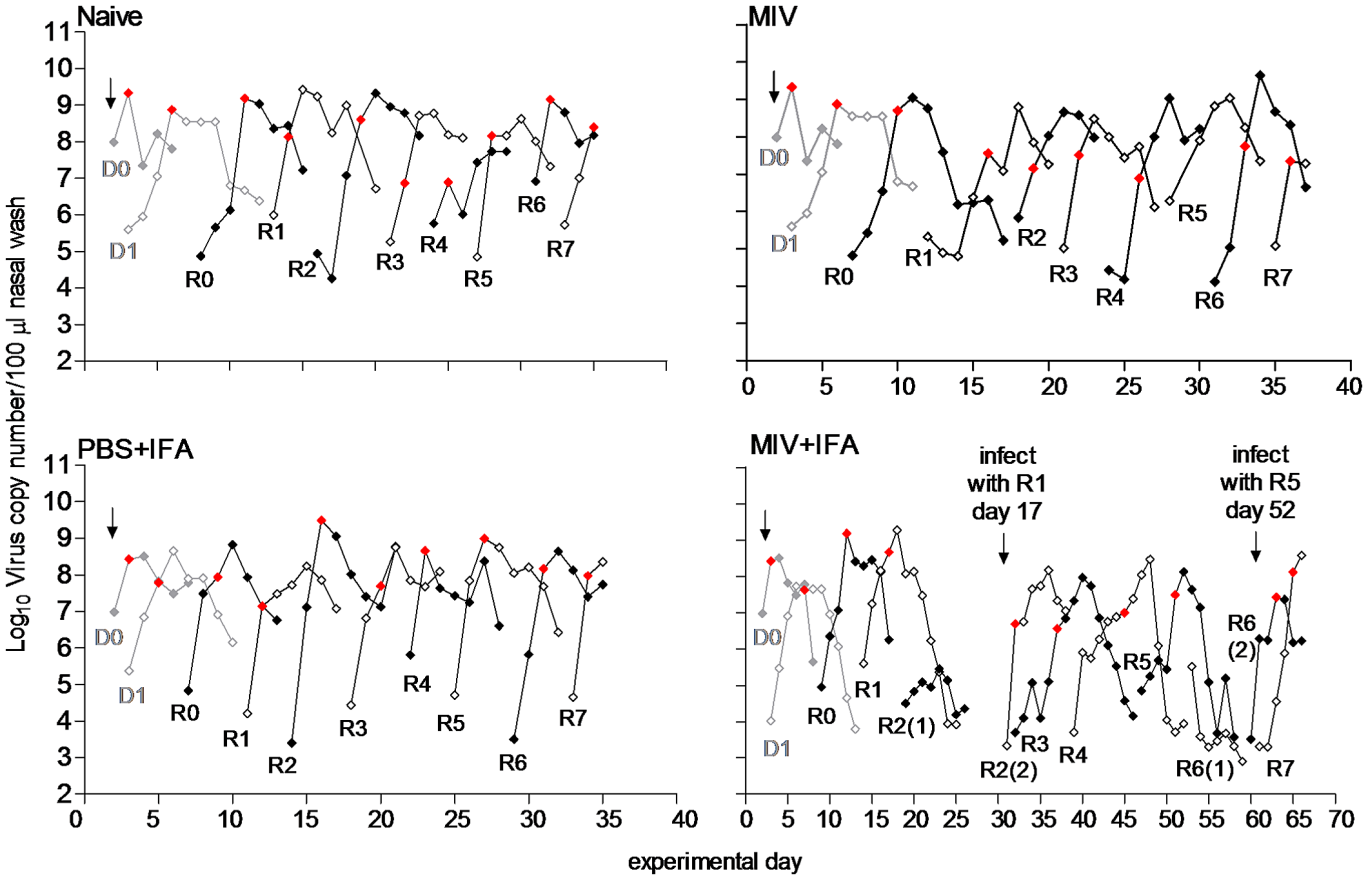
Time course of influenza transmission through passage lines. Naïve donor 0 (D0) ferrets were directly infected with A/Tasmania/2007/2009 virus and co-housed with naïve donor 1 (D1) ferrets to begin the contact transmission model. D0 and D1 ferrets were not included in any experimental group. Once D1 was infected, D0 was removed and a ferret from one of the four experimental groups was added (R0). Transmission then proceeded through R0–R7 ferrets for each group. Line A of each group is shown. The same D0 and D1 ferrets were used to establish the passage line A of the naïve and MIV experimental groups. The same D0 ferret infected two different D1 ferrets to establish the passage line A of the PBS+IFA and MIV+IFA experimental groups. Nasal washes were collected daily from ferrets and virus load was measured by real time RT-PCR. During the experiment, both the rapid test result (PBS+IFA, MIV+IFA day 0–26) and the raw Ct value (naïve, MIV, MIV+IFA day 27 onwards) were used as a marker of infection and transmission. The data points at which transmission of virus to recipient ferrets was deemed to have occurred are identified as red symbols. Direct intranasal inoculation indicated by arrow.

We examined transmission of A/Tasmania/2004/2009 following intranasal inoculation of naïve ferrets or ferrets immunized with adjuvant and vehicle (PBS+IFA), MIV, or MIV+IFA (**Figure 1** and **Figure S1**). In each passage line, we examined seven transmission events and confirmed effective transmission by detection of virus in nasal washes as described in Materials and Methods. Contact transmission failed twice for both MIV+IFA passage lines (**Figure 1** and **Figure S1**). In the first instance this could be attributed to difficulty in detecting shed virus using an influenza rapid test (**Figure 1** R1–R2(1); **Figure S1** R0–R1(1)), so virus shedding in this group was subsequently assayed by daily real time RT-PCR. Transmission was restarted artificially by direct intranasal inoculation of a new recipient ferret with the nasal wash from the previously infected ferret. The second transmission failure (R5–R6(1)), detected using real time RT-PCR, was not due to high immunization antibody titre (data not shown) and transmission was also restarted as described.

**Figure 2:**
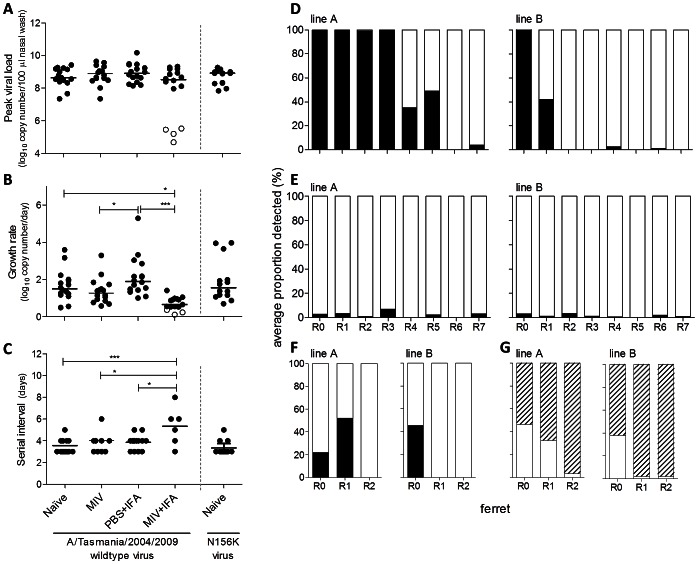
Virus replication and transmission kinetics in ferret passage lines and emergence and persistence of the N156K mutant. (**A–C**) Nasal washes were collected daily from ferrets from passage lines (R0–R7) and assayed for viral RNA by real time RT-PCR as a measure of infection and transmission. (**A**) The peak viral load detected in the nasal wash from each ferret, (**B**) the viral growth rate ((peak virus load – first day virus load)/days to reach peak) and (**C**) the serial interval of virus transmission between ferrets (number of days between detection of infected and infecting animals) were determined for each immunization line. Ferrets artificially infected by intranasal virus inoculation are not included. Ferrets that failed to be naturally infected by contact transmission are indicated in white circles. Statistics do not include ferrets that failed to be naturally infected. (**D**–**E**) The proportion of N156 wildtype (black) and N156K (white) viruses in peak day nasal wash samples from all ferrets from the MIV+IFA (**D**) and N156K naïve (**E**) passage lines quantified by pyrosequencing. (**F–G**) Mixtures of (**F**) N156 wildtype and N156K or (**G**) N156K and K142N+N156K viruses were passaged by contact transmission through naïve ferrets. The proportion of N156 wildtype (black), N156K (white) or K142N+N156K (striped) in peak day nasal wash samples was quantified by pyrosequencing.

The virus kinetics in all passage lines was assessed using real time RT-PCR to determine the relative copy number. There was no difference in the peak viral load achieved in naturally infected ferrets from any of the groups (**Figure 2A**). However growth rate of virus in the ferrets from the MIV+IFA passage lines was significantly lower compared to naïve and PBS+IFA-immunized ferrets (−0.83 log_10_ copy number/100 µl/day (*p = 0.049*) and −1.3 log_10_ copy number/100 µl/day (*p<0.001*), respectively). The growth rate of virus in the ferrets from the MIV passage line was also significantly lower compared to the PBS+IFA-immunized ferrets (−0.77 log_10_ copy number/100 µl/day (*p = 0.049*)) (**Figure 2B**). The time between transmission events (serial interval) was longer for the MIV+IFA line (5.3 days) compared to all other lines (naïve 3.6 days (*p = 0.001*); PBS+IFA 3.8 days (*p = 0.010*); MIV 3.9 days (*p = 0.011*)) (**Figure 2C**). Within each passage line, there was no significant change in any of the kinetics measurements as passaging progressed, suggesting that the growth characteristics and transmissibility of the virus remained stable over time (data not shown).

### Emergence and persistence of a mutation in the HA1 protein in a known antigenic site following passaging of A(H1N1)pdm09 virus in immunized ferrets

RNA was extracted from nasal wash samples from the peak day of virus shedding and infection (as determined by real time RT-PCR) for genetic analysis. Once a mutation appeared in the HA or NA proteins from a ferret of any passage line, it persisted and was transmitted to all subsequent ferrets. Following inoculation of donor ferrets (D0) with egg-grown A/Tasmania/2004/2009, we detected amino acid substitutions in the HA at residues 191 (L191I) and 223 (R223Q) (**Table 2**). The same mutations were also detected following passage of another egg-grown A(H1N1)pdm09 virus (A/Auckland/1/2009) in ferrets (data not shown). Of interest, mutations that alter residues 191 and 223 have been associated with host specificity and receptor binding [28], [29].

Additional mutations that became fixed in the HA protein were specific to each passage line. Mutations arose in the naïve passage lines (G131S, D187E, **Table 2**) and in the MIV passage line (D187V, **Table 2**). G131S creates a potential glycosylation site and D187 has been associated with host specificity [30], [31]. The G131S, D187E and D187V viruses were antigenically indistinguishable from the wildtype virus in HI assays with serum from ferrets infected with egg-grown A/Tasmania/2004/2009 (data not shown). D14E, identified in one of the MIV+IFA passage lines, lies outside predicted antigenic sites [31], does not alter glycosylation and was not tested further.

**Figure 3:**
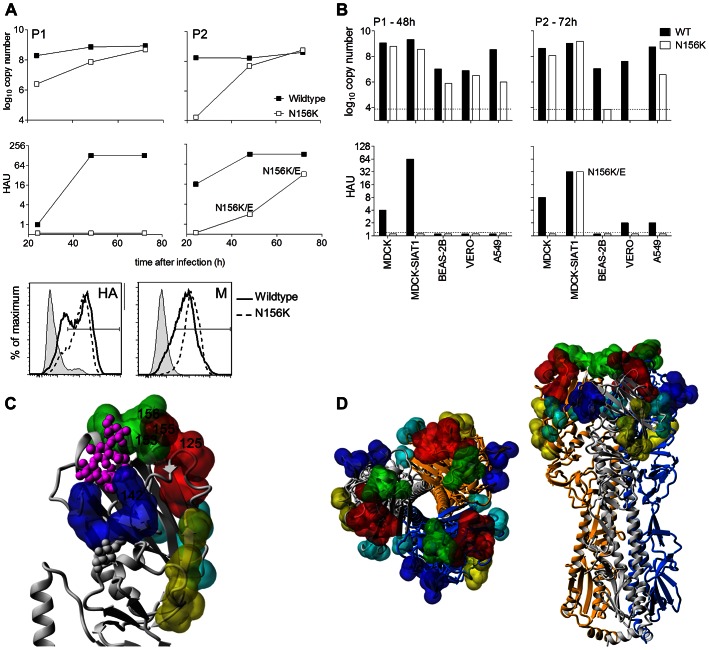
Growth, detection and adaptation of N156K virus in cell cultures. (**A**) Virus underwent two passages in MDCK-SIAT1 cells (P1 and P2). Supernatant was collected during both passages, at 24, 48 and 72 h, and virus load was quantified by real time RT-PCR and hemagglutination (HAU). Infected cells were also harvested at 48 h and surface HA and M protein expression measured by flow cytometry. Cell culture adaptations detected by sequencing in the HA protein are shown in each graph. No adaptations in NA were detected. (**B**) Virus underwent two passages (P1 and P2) in the indicated cell lines. Virus load was quantified in supernatant as above, and cell culture adaptation was detected by sequencing the HA protein as indicated. Limit of detection is 10^3.8^ copies. (**C**) Location of HA mutations in antigenic regions identified in this study. Visualization of mutation positions relative to classical antigenic sites [6] are shown as colored bubbles (Sa – red; Sb – green; Ca1 – cyan; Ca2 – blue; Cb – yellow). Pink balls indicate a bound host receptor ligand (α-2,6-linked); white balls indicate sugars on glycosylation sites. (**D**) Location of antigenic sites in HA trimer, top and side view, respectively.

The N156K mutation arose in both passage lines of MIV+IFA-immunized ferrets (**Table 2**). Position 156 of HA is in antigenic site Sb [6] (Sa in [31]) (**Figure 3C, D**). The relative proportions of the N156K mutant and wildtype N156 viruses were quantified in samples from both passage lines of MIV+IFA-immunized ferrets using a pyrosequencing assay. The N156K mutant emerged in R4 of line A, persisted at a similar proportion in R5, and became dominant by R6. In line B, the N156K mutant emerged earlier, in R1, and became dominant in R2, persisting through to R7 (**Figure 2D**). The emergence of the N156K mutation did not result in a change of virus kinetics (peak viral load, growth rate or serial interval) compared to N156 wildtype virus (data not shown).

To confirm the pyrosequencing data and to determine whether the N156K mutation was present in the original inoculum, the proportion of the N156K mutant was quantified by cloning and sequencing the HA1 gene from the original inoculum (n = 480) and from samples from the R7 ferret in each passage line, and the ferrets in which the N156K mutation was first detected (MIV+IFA R4, line A and R1, line B) (n = 96 for each) (**Table S1**). N156K was detected only in virus samples collected from ferrets in the MIV+IFA immunization group; proportions were similar by cloning and pyrosequencing assay (**Figure 2D** and **Table S1**). All mutations detected in the original sequencing were also detected by cloning. Cloning revealed that L191 and R223 were mixed populations (L191L/I and R223R/Q) in the original inoculum.

Two mutations were detected in the NA protein: M15I emerged in the MIV passage line A and I46F in the MIV+IFA passage line B (**Table 2**). Residue 15 is located in the N-terminus, while residue 46 is located in the stalk of the NA, therefore both substitutions are well outside the enzymatic site of the NA protein [32]. Full genome sequencing of R7 samples from all passage lines detected isolated changes in some genes (e.g. M, NP, PB1, PB2, PA), but none of these substitutions were in known determinants of virulence and/or transmission (**Table 2**).

### N156K A/Tasmania/2004/2009 HA mutant virus is fit in naïve ferrets

To assess the ability of the N156K mutant virus to transmit and persist *in vivo*, N156K variant viruses (from MIV+IFA R7 ferrets) were passaged a further seven times by contact transmission in naïve ferrets (passage lines N156K naïve A and B). The serial interval between transmission events was similar for wildtype and N156K virus passaged in naïve ferrets (**Figure 2C**). Transmission proceeded without interruption (**Figure S2**) and both viral growth rate (**Figure 2B**) and peak viral loads (**Figure 2A**) were similar. Genetic analysis found no changes in the HA protein of virus isolated from the R7 ferret of N156K line A compared to inoculum (D14E+N156K). However, an additional mutation was detected (K142N in R4) and became fixed in the HA of passage line B (K142N+N156K, **Table 2**). Position 142 of HA is in antigenic site Ca2 [6], [31] (**Figure 3C, D**). K142N is not predicted to alter glycosylation. Full genome analysis found no other significant changes (**Table 2**). In both lines, pyrosequencing and sequencing of clones showed N156K persisted at approximately 100% in each sample and did not revert to wildtype N156 (**Figure 2E** **,** **Tables 2** **, S1**). The introduction of the K142N mutation did not result in a change of virus kinetics peak viral load, growth rate or serial interval) compared to N156K alone virus (data not shown).

To directly compare the fitness of the wildtype, N156K and K142N+N156K viruses in the absence of immune pressure [33], naïve ferrets were infected with mixtures of either N156 wildtype and N156K viruses (42∶58% ratio from line B R1 (**Figure 2D**, and **Table S1**, MIV+IFA B R1)) or N156K and K142N+N156K viruses (76∶24% ratio by pyrosequencing). Directly infected naïve ferrets were co-housed with naïve recipients and two transmission events occurred as before. The N156K virus outgrew the N156 wildtype virus after one or two transmission events, in duplicate experiments (**Figure 2F**), as in the original experiment (**Figure 2D**). The K142N+N156K virus outgrew the N156K virus after one or two transmission events (**Figure 2G**). This suggests that the N156K variant fitter than the N156 wildtype virus in the ferret model. It also suggests that the K142N mutation, when added to N156K, gives a selective advantage. However, as the K142N mutation was acquired in only one of the two lines of passaging the N156K virus seven times in naïve ferrets (**Table S1**), it is not essential for replication and transmission in the ferret model.

### The N156K A/Tasmania/2004/2009 HA mutant virus does not agglutinate red blood cells and adapts rapidly *in vitro*

The HA of human influenza viruses preferentially interacts with receptors terminated with Neu5Acα-2,6-linked to galactose (reviewed in [34], [35]) and ferret lung epithelial cells predominantly express α-2,6-linked Neu5Ac residues [21]. As MDCK-SIAT1 cells have enhanced levels of α-2,6-linked Neu5Ac receptors [36], as confirmed by flow cytometry (data not shown), this cell line was used first for virus isolation. Isolates from naïve, PBS+IFA and MIV passage lines were detected in culture supernatants at high titres by agglutination of turkey RBC (HA>128). However, isolates from ferrets of the MIV+IFA and N156K naïve passage lines (herein referred to as N156K mutant virus) had only low hemagglutination titres (HA≤4). Real time RT-PCR for the matrix gene indicated that levels of viral RNA in the N156K mutant virus culture supernatant at 72 h post-infection were similar to those in control samples from naïve or PBS+IFA-immunized ferrets (herein referred to as wildtype viruses) (**Figure 3A**, P1). HA and matrix protein were also detected on the surface of N156K-infected cells (**Figure 3A**) and CPE was visible (data not shown), indicating that N156K mutant virus was present, even in the absence of RBC agglutination. Use of RBC from other species (rat, ferret, guinea pig, sheep), or resialylated RBC, or concentrated culture supernatant did not improve sensitivity in hemagglutination assays (data not shown). Upon further passage in MDCK-SIAT1 cells, the N156K mutant virus was detected only at high HA titres (HA≥16) when additional HA mutations were present (**Figure 3A**, P2). As folding of the mutated HA protein may be temperature dependent [37], [38], infection and culture of MDCK-SIAT1 cells with N156K mutant viruses was also attempted at different temperatures (33, 35 and 37°C), yet similar cell culture adaptations in the HA protein were observed (data not shown). Infection and culture were also attempted in the presence of exogenous neuraminidase or oseltamivir carboxylate as changes in HA have been shown to affect the HA/NA balance and influence hemagglutination and isolation in different *in vitro* culture systems [39]. N156K was not able to be isolated without culture adaptations in the presence of exogenous neuraminidase, or oseltamivir carboxylate (data not shown). Addition of oseltamivir carboxylate did not improve hemagglutination titres for N156K either (data not shown), indicating no binding effect of the NA [39].

Culture adaptations of the HA protein were detected at residues 153, 155 and 156, most commonly as a mixed population at position 156 (N156K/E) or with mutations at positions 153 or 155 in addition to N156K (K153E+N156K or G155E+N156K) (**Figure 3A** **,** **Table 3**). Culture adaptations were also seen in antigenic sites Sa and Sb (**Figure 3C, D**). These adaptations were not detected by clonal analysis in the nasal washes from R7 ferrets of any passage line, in the R4 or R1 ferrets of MIV+IFA passage lines A and B respectively, or in the original virus inoculum (**Table S1**). Culture of virus from peak day samples containing mixed populations of wildtype (N156) and N156K viruses resulted in outgrowth of the wildtype N156 virus as determined by RNA extraction and sequencing (data not shown). No MDCK-SIAT1 culture adaptations were detected in the NA gene.

**Table 3:** Mutations and cell culture adaptations in A(H1N1)pdm09 HA protein.

Data source	Passage conditions	Mutations or Culture Adaptations (H1 numbering, without signal peptide)	125	142	153	154	155	156
		H1 numbering, with signal peptide	142	159	170	171	172	173
		H3 numbering, without signal peptide	128	145	156	157	158	159
Current study	A/Tasmania/2004/2009 inoculum	n/a	N	K	K	K	G	N
	Ferret nasal wash, MIV+IFA passage lines	(D14E+) N156K mutant						K
	Ferret nasal wash, N156K naïve passage line B	K142N+N156K mutant		N				K
	MDCK-SIAT1 passage of ferret nasal wash	G155E+N156K^a^		(N)^a^			E	K
		G155E+N156K/E^a^		(N)^a^			E	K/E
		N156E^a^		(N)^a^				E
		N156K/E^a^		(N)^a^				K/E
		K153E+N156K		(N)^a^	E			K
		K153E+N156E^a^		(N)^a^	E			E
		K153E+N156K/E^a^		(N)^a^	E			K/E
Published research *in vitro* studies	MDCK re-passage of A(H1N1)pdm09 reassortant rescued virus [29]	K153E			E			
		K154E				E		
		G155E^b^					E^b^	
	MDCK passage of A(H1N1)pdm09 wildtype virus with human mAb [15]	K154N+S183P				N		
		G155E					E	
	Egg passage of A(H1N1)pdm09 reassortant virus with mouse mAb [16]	K153E (alone or co-mutation with N125D)	D		E			
		G155E (alone or co-mutation with D187N, D187E)					E	
		N156D (alone or co-mutation with N125S or D187N)	S					D
		K153E+G155E			E		E	
		G155E+N156D					E	D
Surveillance studies	Surveillance of human influenza original clinical specimen [40]	N125D+N156K	D					K
	Melbourne WHO CC surveillance original samples	A/Victoria/906/2010 (N125D+N156K)	D					K
		A/Tasmania/16/2011 (N156K)						K
		A/Wellington/94/2010 (N125D+N156K)	D					K
	Melbourne WHO CC surveillance MDCK isolates^c^	A/Victoria/906/2010 (N125D+N156K/E)	D					K/E
		A/Tasmania/16/2011 (N156N/K/D/E)						N/K/D/E
		A/Wellington/94/2010(N125D+N156E)	D					E

a Also occurred with K142N+N156K.

b Introduced into virus, not isolated in culture experiments.

c Sequences available on GISAID.

Attempts to isolate the N156K mutant virus in a variety of other cell lines (MDCK, Beas-2B, Vero, A549) as well as embryonated hen's eggs, were unsuccessful. The virus either did not grow, or it grew and was detected by real time RT-PCR, but rapidly adapted in the HA at amino acids 153–156 (**Figure 3B**).

Attempts were also made to purify virus by ultracentrifugation from ferret bronchoalveolar lavage and homogenized lung samples, but this did not yield sufficient virus for analysis.

### Detection of N156K and culture adaptations in influenza surveillance data

The N156K mutation has been detected in human A(H1N1)pdm09 clinical samples (analyzed for surveillance purposes at the WHO Collaborating Centre for Reference and Research on Influenza, Melbourne), both alone and in conjunction with N125D, which is located in antigenic site Sa [6], [40] ) (**Table 3** and **Figure 3C, D**). N156D has been detected following passage of A(H1N1)pdm09 virus in embryonated hen's eggs under immune pressure [16]. Mutations at G155 and K153 have been reported following *in vitro* passage of A(H1N1)pdm09 viruses in the absence [29] and presence of monoclonal antibodies (mAbs) [15], [16]. Cell culture adaptations have also been detected in routine surveillance at the WHO Collaborating Centre for Influenza, Melbourne, when passaging A(H1N1)pdm09 viruses with mutations at N156 (**Table 3**). Analysis of all A(H1N1)pdm09 sequences from 2009–2012 in GISAID and GenBank found N156K, K153E and N156D at similar low frequencies (0.15, 0.20 and 0.27%, respectively); G155E has been reported more often (1.30%, **Table 4**). Among 8446 A(H1N1)pdm09 HA sequences with classifiable passage information, most were MDCK cell isolates (69%) followed by original clinical samples (18%), MDCK-SIAT1 isolates (8%) and embryonated hen's egg isolates (5%). There is no change in isolation patterns in each year (2009–2012, data not shown). The N156K mutation was found more commonly in original specimens than in cell isolates. In contrast, K153E and G155E adaptations were strongly associated with growth in MDCK cells (**Table 4**).

**Table 4:** Analysis of passage history of surveillance viruses with mutations in key residues of HA1 as reported on GISAID and GenBank.

				Passage history Odds Ratio (mutant vs wildtype)			
Mutation	No. reported^a^	No. isolates (mutant/wildtype)^b^	Total frequency (%)^c^	Egg isolate	MDCK cell isolate	MDCK-SIAT1 cell isolate	Original clinical sample
N125D	581	321/7730	3.99	2.05	0.96	0.57	1.04
K153E	19	16/8018	0.20	1.59	6.55	-	-
G155E	133	103/7827	1.30	0.46	4.63	0.68	0.05
N156D	31	22/7980	0.27	-	4.32	0.52	0.22
N156K	22	12/7980	0.15	-	0.43	1.00	3.28
L191I	47	25/8008	0.31	55.31	0.20	-	-
Q223R	133	71/7966	0.88	564.42	0.02	0.00	0.00

The odds ratio, indicating strength of association to passage history, for mutant versus wildtype virus is indicated. Mutations with <10 samples with any passage information were omitted (e.g. K156E). (-) indicates that 10–30 records with passage information were available, and no reports were indicated in this passage history.

a Occurrence of mutation in all 16740 A(H1N1)pdm09 sequences on GISAID and/or Genbank, regardless of passage history up to December 2012.

b Occurrence of mutant or wildtype in all A(H1N1)pdm09 sequences on GISAID with passage history information.

c % Occurrence of mutant in all A(H1N1)pdm09 sequences on GISAID with passage history information.

### Cell culture adaptations may disguise the effect of N156K on the antigenic profile of A/Tasmania/2004/2009 virus

Other *in vitro* studies have demonstrated that mutations at amino acids 153, 155 and 156 of A(H1N1)pdm09 HA1 result in antigenic drift with a reduction in HI titre to the mAbs used to induce changes in the antigenic sites [15], [16] and to serum from wildtype A(H1N1)pdm09-infected ferrets [29]. Adaptations K153E and G155E are also antigenically distinct from each other [16]. We generated viruses containing the HA mutation(s) detected in our experiments by reverse genetics. All reverse genetics viruses used contained the ferret-specific adaptations L191I and R223Q in the HA protein. Neither the N156K alone nor K142N+N156K virus could be rescued as a pure population without adaptation. Rescues were attempted with both an A(H1N1) (A/PuertoRico/8/1934) and an A(H1N1)pdm09 (A/Perth/261/2009) complete backbone and were also attempted with ‘humanized’ vectors containing receptors L191 and Q223, but neither was successful (data not shown). Antigenic analysis by HI assay showed a reduction in HI titre for all reverse genetics viruses with mutations at positions 153–156, against serum from a ferret infected with the original inoculum of A/Tasmania/2004/2009 virus, compared to wildtype virus. Both reverse genetics mutant N156E as well as surveillance viruses with mutations and adaptations at N156 showed reduced HI titre compared to wildtype virus, indicating the antigenic importance of amino acid 156. The K142N mutation had no effect (**Table 5**).

**Table 5:** Antigenic analysis of mutations in the HA protein from amino acid 153–156.

Hemagglutination Inhibition titre			Sera	
	Virus source and passage history	Virus	Wildtype^a^	MIV+IFA^b^
Virus	Reverse genetics ferret-adapted A/Tasmania/2004/2009 HA (L191I, R223Q) and NA, MDCK-SIAT1 passaged^c^	Wildtype	**5120**	640
		K153E	160	160
		K153E+N156K	160	160
		G155E	160	160
		G155E+N156K	160	160
		N156E	320	160
		K153E+N156E	320	160
		G155E+N156E	80	160
		K142N	10240	640
		K142N+K153E+N156K	160	20
		K142N+G155E+N156K	80	20
		K142N+N156E	160	40
		K142N+G155E+N156E	80	20
	Egg passaged	A/Tasmania/2004/2009	5120	640
	Egg passaged, split	Monovalent human vaccine	10240	**640**
	MDCK-SIAT1 passage	A/Tasmania/2004/2009	5120	320
	Surveillance A(H1N1)pdm09 viruses with mutation at antigenic sites of HA, MDCK passage	A/Victoria/906/2010 (N125D, N156K/E)	320	320
		A/Tasmania/16/2011 (N156N/K/D/E)	160	80
		A/Wellington/94/2010 (N125D, N156E)	160	160

Homologous titre (bold) is indicated.

a Ferrets were infected with egg-grown A/Tasmania/200/2009 virus.

b Serum following a single immunization with MIV+IFA.

c N156K reverse genetics virus is not included as it could not be rescued.

All reverse genetics viruses were also tested against serum from a ferret immunized once with MIV+IFA. All viruses containing mutations at positions 153–156 showed a four-fold reduction in titre compared to wildtype virus. Although the K142N mutation on its own had no effect, addition of this mutation to viruses with mutation/s at positions 153–156 reduced their titre at least four-fold further (**Table 5**).

### The HA antigenicity and receptor binding profile are altered in the N156K A/Tasmania/2004/2009 HA mutant virus

Our observation that N156K mutant viruses were not detected by hemagglutination and did not persist without adaptation *in vitro* suggested an alteration in receptor binding specificity. N156K and K142N (and K153E, R223Q) are located in regions on the HA protein proposed to contact sugars beyond Neu5Ac on cell surface receptors [28]. Thus we hypothesized that these mutations altered not only antigenicity but also receptor binding compared to wildtype virus.

**Figure 4:**
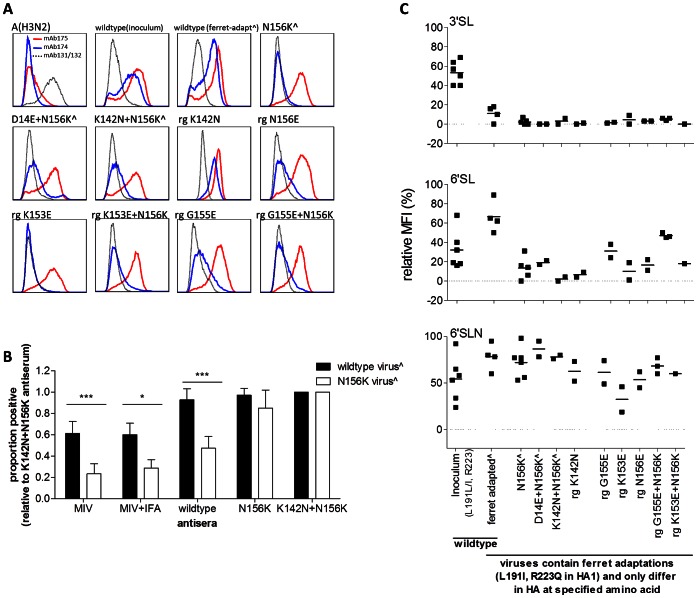
Analysis of antigenicity and glycan binding to HA on influenza virus-infected cells. MDCK-SIAT1 cells were inoculated with A/Tasmania/2004/2009 egg-grown inoculum, ferret nasal wash (indicated by ∧) or rescued reverse genetics ferret-adapted viruses (indicated by ‘rg’) or a control A(H3N2) egg-grown virus. After 48 h, cells were analysed for binding of (**A**) anti-HA mAbs, (**B**) post-immunization ferret antisera (MIV or MIV+IFA) or post-infection ferret antisera (wildtype A/Tasmania/2004/2009, N156K or K142N+N156K virus) or (**C**) synthetic glycans. (**B**) To control for differences in infection rate between wildtype and N156K viruses, antibody binding is expressed as the proportion relative to K142N+N156K antiserum (% positive for test antiserum/% positive K142N+N156K antiserum). Mean+standard deviation of six paired experiments are shown for all antisera except MIV+IFA which is calculated from three paired experiments. (**C**) Glycan binding is expressed as a proportion to itself ((mean fluorescent intensity (MFI) with glycan-MFI without glycan)/MFI with glycan×100), for influenza A matrix positive cells only. Individual experiments are shown by each symbol.

HA structure was investigated by flow cytometry [41] using mAb174 and mAb175, which are specific for the Sa/Sb (K153 and G155) and Cb (T72) antigenic regions of the A(H1N1)pdm09 virus HA, respectively. All A(H1N1)pdm09 wildtype and variant viruses were recognized by mAb175 (**Figure 4A**). The A/Tasmania/2004/2009 original inoculum and ferret-adapted wildtype viruses were recognized by mAb174, but binding was greatly reduced in the presence of the N156K mutation and cell culture adaptations (**Figure 4A**). This effect was specific for viruses with mutations in the Sa/Sb antigenic region as the virus with K142N mutation alone was still recognized by mAb174.

Direct binding assays using antisera from immunized and virus-infected ferrets were used to assess the antigenicity of the N156K mutant virus. Antisera from MIV−, MIV+IFA-immunized ferrets or from ferrets infected with wildtype virus recognized cells infected with N156K virus less efficiently than cells infected with wildtype virus. In contrast, post-infection antisera from ferrets infected with N156K or K142N+N156K viruses bound cells infected with wildtype or N156K mutant virus to a similar degree (**Figure 4B**). These data suggest that the N156K virus is antigenically distinct from wildtype virus. Furthermore, it is likely that the wildtype antiserum contains a high level of antibodies directed towards the N156 region of the HA protein, whilst the N156K and K142N+N156K antisera contains antibodies that are directed towards a region of the HA protein, common to both wildtype and N156K HA.

HA receptor specificity was assessed by binding of synthetic multivalent glycans. Human influenza viruses have been shown to bind predominantly to 6′ sialyllactose (6′SL) and 6′ sialyllactosamine (6′SLN) and, to a lesser extent, to 3′ sialyllactose (3′SL), as representatives of α-2,6- and α-2,3-linked Neu5Ac receptors [28], [41]–[45]. HA on the surface of cells infected with ferret adapted A/Tasmania/2004/2009 wildtype virus bound to both 6′SL and 6′SLN (**Figure 4C**). Binding was more restricted compared to the original egg inoculum virus, presumably due to the mutations L191I and R223Q (**Figure 4C**). Introduction of the N156K mutation to the ferret adapted virus resulted in binding to only 6′SLN, indicating a strong preference for α-2,6-linked Neu5Ac receptors containing GlcNAc rather than galactose (**Figure 4C**, N156K, D14E+N156K, K142N+N156K). K142N and N156E displayed a similar profile to N156K. K153E showed weak binding to 6′SLN, G155E showed binding to 6′SL and 6′SLN. Addition of G155E to N156K increased 6′SL binding, while the addition of K153E to N156K did not alter the binding preference.

### Predicted receptor binding properties and structural analysis using 3-D modeling

Computer modeling with α-2,6- and α-2,3-linked Neu5Ac receptors was performed to enable a structural basis for the N156K mutation and subsequent cell culture adaptations. The computational results were also compared to the glycan binding results (**Figure S3A**). N156K is at the interface of three previously crystallized antibody binding sites [46]–[48] (**Figure S3B**). Since amino acid mutations that involve charge changes can alter the strength of the electromagnetic field around the HA globular head, and long range electrostatic forces are often a first step to facilitate interaction with ligands and other proteins, changes in the electrostatic surface potential due to the observed mutations were also analyzed.

**Figure 5:**
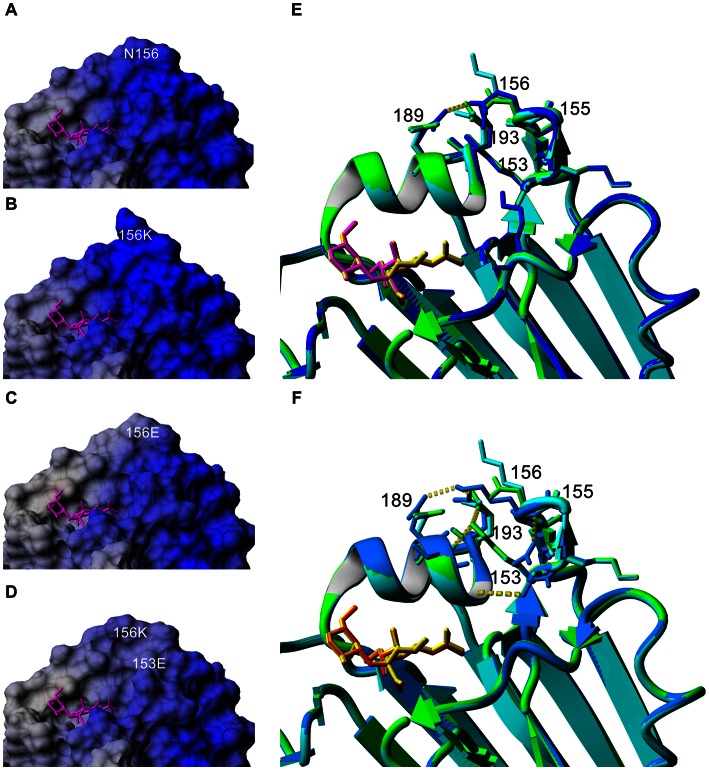
3-D modeling of HA containing N156K and cell culture adaptations. (**A–D**) Comparison of electrostatic surface potential of wildtype and single and pair mutations in the HA head domain, calculated with the Particle Mesh Ewald method implemented in YASARA. Blue indicates positive and red indicates negative charge potential. A host receptor analogue is shown in magenta. (**E,F**) Structural modeling of single and pair mutations in HA with bound α-2,6- or α-2,3-linked host receptor ligands. (**E**) Model of N156 wildtype (green HA/yellow ligand), N156K (cyan HA/red ligand) and N156E (blue HA/purple ligand) shown with α-2,3 host receptor analogue. (**F**) Comparison of N156 wildtype (green HA/yellow ligand) and N156K (cyan HA/red ligand) with double mutant K153E+N156K (light blue HA/orange ligand).

Although position 156 is not in the receptor binding site, our analysis suggests that small changes at this position influence HA binding, stability and receptor specificity. The N156K mutation noticeably adds positive charge potential compared to wildtype HA (**Figure 5A, B**). In contrast, N156E significantly reconfigures the distribution of charge (**Figure 5C**). N156K is predicted to prefer α-2,6-linked receptors (**Figure 5E**), whilst in our models it is more favorable for the N156E mutation to bind to α-2,3-linked receptors (**Figure 5E**). Adding K153E to N156K reduces the positive electrostatic potential of the HA head domain (**Figure 5D**) as does the addition of G155E to N156K (**Figure S3C**). The addition of K153E to N156K (and G155E to N156K) appears compensatory, allowing stronger binding to α-2,3 receptors compared to N156K alone (**Figure 5F**, **Figure S3C,D**). However, this increased α-2,3 receptor binding was not evident in the glycan binding studies that were undertaken (**Figure 4C**).

## Discussion

This study is the first to describe the emergence of an A(H1N1)pdm09 escape variant, with altered receptor binding, following vaccine-induced immune pressure in a ferret model of contact transmission. This N156K mutation was generated in two independent lines of MIV+IFA-immunized ferrets, but was not detected in the virus used to infect ferrets, nor did it appear in unimmunized ferrets, suggesting that the N156K mutation had a selective advantage when placed under non-sterilizing immune pressure.

Virus fitness was investigated in the presence and absence of immune pressure in this study. The N156K virus transmitted between immunized ferrets for up to four passages, although a break in transmission occurred in both lines. A break in transmission in vaccinated animals has also been noted in an artificial infection system of a laboratory influenza strain in mice [13]. The investigation of virus kinetics under immune pressure is unique to this ferret study. The emergence of the N156K virus did not alter the virus kinetics in the MIV+IFA-immunised ferrets. There was no change in peak viral load, growth rate or serial interval between transmissions. This is likely a reflection of the *in vivo* model system. Adjuvant has been shown to restrict and broaden the immune response (reviewed by [49]), yet was necessary to induce even a detectable immune response in the ferret model [24]. The influence of a different adjuvant, or even a background of immunological experience through previous infection(s), may warrant further investigation. When passaged in naïve ferrets, the growth kinetics and transmission potential of the N156K virus was equivalent to the wildtype virus, indicating that the N156K mutation was not deleterious in non-immune ferrets. The N156K virus outgrew the wildtype virus in both naïve and immunized ferrets when in direct competition, suggesting that the N156K mutant is fitter than the wildtype virus in both the presence and absence of immune pressure.

The interplay between antigenic drift and receptor binding specificity and affinity has been well studied for the HA of influenza viruses [13], [16], [50]. Due to the close proximity of the antigenic sites to the receptor binding site, antigenic changes are often accompanied by changes in the receptor binding properties of the virus [13], [15], [16] and changes in receptor binding properties can also produce antigenic variants [13]. Passage of influenza virus under immune pressure *in vivo* may favor mutations that facilitate antibody escape, but may also increase viral HA avidity for cell surface receptors. Subsequent passaging in the absence of immune pressure *in vivo* may induce compensatory mutations that reduce avidity and can affect antigenicity [13]. Furthermore, mutations in A(H1N1)pdm09 HA that change positive electrostatic charge have been associated with changes in the affinity for Neu5Ac receptors [16]. We have demonstrated that the N156K mutation produced structural changes that altered receptor specificity, including the loss of ability to agglutinate RBC from a variety of species. Changes in hemagglutination patterns with different species' RBC have also been associated with antigenic drift of A(H3N2) viruses [43], [50]. The N156K mutation also increases the positive charge potential, which may be important for surface interactions of the HA protein with antibodies or more remote carbohydrate moieties extending from the natural host receptor structure. The cell culture adaptations, K153E and G155E, are predicted to induce structural changes in the HA head that reduce the positive electrostatic potential of the HA head domain. These changes have been shown to reduce viral HA receptor binding avidity for α-2,3- and α-2,6-linked Neu5Ac receptors *in vitro* [16]. Thus, we hypothesize that the N156K mutation increases receptor binding avidity compared to wildtype virus. When cultured *in vitro*, the cell culture adaptations negate the significant effects of the N156K mutation on the charge distribution and alter the receptor binding preference of the HA protein (and possibly the avidity), which facilitates hemagglutination. Attempts to assess the avidity of the N156K and K142N+N156K mutants were unsuccessful due to virus isolation difficulties and the inability of the viruses to bind RBC. Interestingly subsequent passage of the N156K mutant virus in naïve ferrets did result in an additional mutation in the HA protein, K142N, in one passage line. Although this double mutant outgrew the N156K mutant in a competitive mixture *in vivo*, the K142N mutation was not acquired in both lines of passaging suggesting it is not essential for transmission in naïve ferrets. K142N did not alter receptor binding specificity in the assays used in this study, but whether changes in avidity have occurred is unknown. Overall, these data suggest that selection of the N156K mutant, maintained through contact transmission in ferrets, drives emergence of a virus with altered, but effective, binding to receptors of the ferret respiratory tract, and is capable of extensive replication *in vivo*. *In vitro* isolation difficulties indicate more detailed analysis of receptors in the ferret respiratory tract and on cell lines used for routine isolation of influenza virus may be needed. As antiserum from a wildtype virus-infected ferret appeared directed to the N156 region and could no longer recognise the N156K mutant virus, and antiserum from a N156K virus-infected ferret appeared directed to a different region of the HA common to wildtype and N156K mutant virus, we hypothesize that immunity is directional and drives antigenic drift in this contact transmission model. We anticipate that further passage of the N156K mutant virus in homologous vaccinated ferrets (i.e vaccinated with the N156K mutant) would drive further drift of the A(H1N1)pdm09 N156K virus that would be again accompanied by changes in the receptor binding properties.

Although a pure population of the N156K mutant virus could not be efficiently isolated, either from ferret nasal washes or by reverse genetics, flow cytometry antibody binding assays demonstrated that the N156K mutation causes antigenic escape. This is supported by the following additional evidence: The N156K mutant arose only in ferrets with specific antibodies, and was present in both passage lines. The N156K mutant virus could not be recognized by a monoclonal antibody (mAb174) which drives escape in the Sa/Sb antigenic site of wildtype virus. N156K is computationally predicted to be structurally significant by introduction of a longer charged side chain that changes the distribution of the charge of the HA. Analysis of influenza vaccine strains over time has demonstrated that charge mutations also contribute to antigenic drift (unpublished data, S. Maurer-Stroh and R.T.C. Lee). Alternate mutations at this position, N156E and N156D, have been shown to cause antigenic changes [16], [51]. Other published *in vitro* studies have found mutations at similar sites in the HA protein of A(H1N1)pdm09 viruses ( amino acids 143–146, 148, 153–156, 204) [15], [16], [29], [40] which also affected antigenicity; however interpretation of these *in vitro* experiments is complicated by the different culture conditions and selection pressures employed. Changes can also occur at amino acid positions 153 and 155 in the A(H1N1)pdm09 HA without any immune selection pressure [29], suggesting an instability in this region during cell culture. No changes have been reported at 156 in the A(H1N1)pdm09 HA experimentally without immune pressure. However, monoclonal antibodies from humans and mice (this study and [15], [16]), and polyclonal ferret antibodies raised in this study, all targeted similar regions. This suggests that amino acids 153–156 form an immunodominant region of the HA protein and targeting this area results in a significant antigenic change.

A(H1N1)pdm09 viruses with mutations at position 156 have been reported in human surveillance studies ([40], WHO Collaborating Centre for Reference and Research on Influenza, Melbourne), although they occur relatively rarely (0.15% of all samples on GISAID with passage information). We have demonstrated that this mutation from ferret respiratory samples does not persist in cell culture without further adaptation, or reversion to wildtype virus in mixed populations. The low HA titre in the initial passage, and negative association with cell culture, may prevent identification of original clinical samples containing N156K alone, hence laboratories may exclude these viruses from surveillance studies. Therefore conventional methods for isolation of human samples may select against detection of mutations at position 156. We also cannot discount the possibility that the ferret model has selected for a mutant that has compromised transmissibility in the human population, due to differences in the glycan receptor profiles between the human and ferret respiratory tract, or more complex immunity in humans with their extensive infection and vaccination history. Use of a different A(H1N1)pdm09 strain, cell-cultured virus or an alternate adjuvant/vaccine preparation may have also influenced the mutation profile. Further analysis of viruses from the lungs of MIV-immunized ferrets, where immune escape mutants may also emerge, would be worthwhile.

A(H1N1)pdm09 has circulated over the past four years with limited genetic diversification and no significant antigenic change. Rising population immunity is likely to drive antigenic drift in the A(H1N1)pdm09 virus eventually. Passage in immune ferrets demonstrated that an antigenic mutant can arise in a similar situation. Importantly, antigenic mutations in the HA may also influence receptor binding properties, affecting the efficiency of isolation of viruses for surveillance characterization purposes. Routine genetic analysis of original clinical specimens is therefore important. *In vitro* and/or *in vivo* passage of the 2012/2013 A(H1N1)pdm09 viruses under immune pressure would indicate whether N156K is permissive in the presence of additional mutations or whether other regions of the HA globular head are now under greater selective pressure. Continued examination of influenza virus escape mutants should aid in the identification of future vaccine-breakthrough viruses, enabling their rapid detection through influenza surveillance and ensuring appropriate changes to the A(H1N1)pdm09 component of seasonal influenza vaccine are made in a timely fashion.

## Materials and Methods

### Ferrets

Male and female ferrets (weight, 500–1500 g) were purchased from independent breeders and housed at CSL Limited using services provided under a Support Services Agreement. Serum samples from ferrets were tested by HI assay to ensure seronegativity (titre <20) to currently circulating influenza strains before use. Experiments using ferrets were conducted with approval from the CSL Limited/Pfizer Animal Ethics Committee, in accordance with the Australian Code of Practice for the Care and Use of Animals for Scientific Purposes.

### Viruses and cells

A/Tasmania/2004/2009 virus was isolated from an elderly patient who succumbed to influenza infection in July 2009, as part of routine influenza surveillance activities at the WHO Collaborating Centre for Reference and Research on Influenza, Melbourne. The sample was anonymized. Viruses were passaged in the allantoic cavity of embryonated hen's eggs and MDCK-SIAT1 cells and stored at −80°C. MDCK (CCL-34:ATCC) and MDCK-SIAT1 cells (kindly provided by Hans-Dieter Klenk, University of Marburg, Marburg, Germany) were maintained as previously described [52], in the absence of G418 sulphate. A549 and Vero cells were maintained in the same conditions as MDCK cells. BEAS-2B cells (kindly provided by Dr R. Gualano The University of Melbourne, Melbourne, Australia) were maintained in 1∶1 (v/v) RPMI −1640 (SAFC Biosciences) supplemented with 10% [v/v] FCS, 50 U/ml penicillin, 50 µg/ml streptomycin (SAFC Biosciences): BEGM with BulletKit (Cambrex, Clonetics, USA). Cells were maintained at 37°C, 5% CO_2_ prior to infection and following infection, unless indicated.

### Vaccine formulation and administration

Full or half adult doses (15 µg or 7.5 µg HA in 0.5 mL, respectively) of human monovalent A(H1N1)pdm09 influenza vaccine containing the HA and NA of A/California/7/2009 (MIV) (Panvax, a gift from CSL Limited) were used for immunization. Ferrets were immunized with MIV alone or MIV emulsified in an equal volume [1∶1 (v/v)] Freund's Incomplete Adjuvant (IFA) (Sigma-Aldrich) immediately before administration (1 ml total volume) (MIV+IFA). Control ferrets were immunized with PBS emulsified in IFA (1 ml total volume) (PBS+IFA) or did not receive vaccine (naïve). Adjuvanted vaccines were delivered to anaesthetized animals (0.2 mL/kg, 1∶1 [v/v] Ilium Xylazil-20∶Ketamil, Troy Laboratories) intramuscularly in the quadriceps muscles of both hind legs using a 1-mL syringe with a 22-gauge needle. Animals received one dose of MIV+IFA or PBS+IFA or two doses of MIV two weeks apart. Vaccinated animals were used 24 to 94 days after the final immunization. Serum was collected before and after each vaccination, immediately before ferrets were included in the contact transmission passaging and at sacrifice.

### Virus infection of donor ferrets and passaging of virus by contact transmission

Naïve donor ferrets (termed Donor 0; D0) were anaesthetized and infected artificially by intranasal inoculation with 10^3.5^ 50% tissue culture infectious doses (TCID_50_) egg-grown influenza virus. After inoculation, D0 ferrets were housed individually in a HEPA-filtered isolation unit. The following day, two naïve ferrets (both termed Donor 1; D1) were co-housed with each D0 ferret and remained in continual contact, until they became naturally infected with influenza virus (see below). Once a D1 ferret was found to be infected it was placed in a separate clean cage, to then infect the first ferret of a passage line. Naïve donor ferrets (D0 and D1) were used to establish a transmission chain as previous studies have indicated that direct inoculation of *in vitro* cultured virus can alter viral diversity within a host [53]. Four different D0 ferrets infected seven different D1 ferrets, each D1 ferret was assigned to start one (or two) passage lines (as indicated in **Figure 1** and **S1**). Each line involved sequential passage of virus through eight recipient ferrets that had received one of three different treatments (MIV+IFA, MIV, PBS+IFA) or no treatment (naïve). The first recipient ferrets from each immunization group (R0) were co-housed with one of the D1 ferrets until they became naturally infected. 24 hours after the viral load measurement from each R0 ferret indicated that they were infected, the R0 ferret was co-housed with the next recipient ferret (R1) from their line. Transmission experiments continued for each immunization group until seven passages had been completed (R0 to R7). Duplicate lines were performed for each immunization group, designated line A and line B. Ferrets were sacrificed and respiratory tissues and serum were collected after they had successfully transmitted the virus to the new recipient ferret. The recipient order for the passage lines was randomly assigned in each immunization group, irrespective of HI antibody titre following immunization, or days since immunization.

### Detection of virus infection in ferrets

Nasal washes were collected daily under light sedation (30 mg/kg Ilium Xylazil-20; Troy Laboratories) by delivery of 1 ml PBS supplemented with 50 U/ml penicillin, 50 µg/ml streptomycin (SAFC Biosciences) and 1% w/v BSA (Sigma-Aldrich) into the nostril. Expelled liquid was collected and 140 µl was aliquoted for RNA extraction (see below) and real time RT-PCR analysis, 60 µl for TCID_50_ analysis, 30 µl for rapid test analysis and the remainder stored at ^−^80°C. Animals were weighed and visually inspected daily and their temperature was measured using implanted temperature transponders fitted to identification chips (LifeChip Bio-Thermo, Digivet). Daily nasal wash samples were assessed for virus shedding as a measure of infection by rapid test analysis (BD Directigen EZ Flu A+B kit, Becton Dickinson) or by real time RT-PCR analysis of extracted RNA for matrix 1 gene. Transmission was deemed to have occurred when the new recipient ferret returned a positive rapid test, or a positive real time RT-PCR result (Ct≤25).

### Real time RT-PCR

RNA was extracted from nasal washes, cell culture supernatants or allantoic fluid using the QIAamp Viral RNA Mini Kit (Qiagen). An 8 µl aliquot of RNA was treated with RNAse Free DNase I (New England Biolab) in a total reaction volume of 10 µl, according to the manufacturer's instructions. DNase-treated RNA (4 µl ) was used to amplify influenza A matrix 1 using CDC Atlanta (USA) InfA F primer and InfA probe [54] and modified InfA R primer (5′GGGCATTYTGGACAAAKCGTCTACG3′) with SuperScript III Platinum One-Step qRT-PCR kit (Invitrogen). The RT-PCR consisted of 1 cycle of 50°C for 5 min and 95°C for 2 min and 40 cycles of 95°C for 3 s, 60°C for 30 s using the 7500 Fast Real-Time System (Applied Biosystems) and 7500 Fast System SDS Software version 1.4.0. The threshold was automatically set and Ct determined. RNA standards were prepared from pGEMT-A/California/7/2009 and pGEMT-A/Perth/16/2009 Matrix plasmids using the Riboprobe In Vitro Transcription Systems (Promega). For all runs, samples (assayed in duplicate), no-template control, RNA standards and positive and negative controls were included. The 95% confidence interval for RNA standards was 0.78 Ct (n = 65 assays). The detection limit was 10 copies.

### Preparation of cDNA for genetic analysis

cDNA was synthesized from 5 µl RNA isolated from ferret nasal wash (peak day of infection), cell culture supernatants or allantoic fluid, using the SuperScript III First-Strand Synthesis SuperMix (Invitrogen), with Uni12 primer [55], according to the manufacturer's instructions. cDNA generated from ferret nasal wash was sequenced, used in pyrosequencing assays, or amplified by PCR and cloned.

### Genetic sequencing

Viral genes were amplified from cDNA or DNA plasmids (see below) using MyTaq HS Mix (Bioline) with M13 tagged-gene-specific primers ([56] and WHO Collaborating Centre for Reference and Research on Influenza, Melbourne – sequences available upon request) or plasmid-specific primers, according to the manufacturer's instructions. Unincorporated primers and dNTPs were removed using ExoSAP-IT (Affymetrix), according to the manufacturer's instructions. DNA sequencing was performed with M13 primers (M13F-5′ TGTAAAACGACGGCCAGT and M13R 5′ CAGGAAACAGCTATGACC) or plasmid-specific primers in a 96-well plate format using the BigDye Terminator v3.1 Cycle Sequencing Kit (Life Technologies), followed by the removal of excess dye terminators with a BigDye XTerminator purification kit (Applied Biosystems). The sequence was determined using an automated capillary DNA sequencer (ABI Prism 3500xL). Identification of mixed bases was set at 25%. Sequences were assembled using DNASTAR Lasergene Suite v.9.1.0 Seqman v 9.1.

### Nucleotide sequence accession numbers

The nucleotide sequences for all segments of A/Tasmania/2004/2009 egg-grown virus inoculum are available from the GISAID public database under accession number EPI_ISL_129743. A/California/7/2009 sequences used in this study were from GenBank: CY058519, GQ214336, CY121681, GQ338390, CY121684, CY121685, GQ323558, FJ984387.

### Generation of DNA plasmids

The HA1 gene of A/Tasmania/2004/2009 virus from cDNA generated from ferret nasal wash was amplified using gene-specific primers and cloned into pGEM-T Easy vector (Promega) according to the manufacturer's instructions. For reverse genetics, the entire HA and NA genes of A/Auckland/1/2009 (A(H1N1)pdm09) virus [24] were amplified from cDNA generated from a clinical specimen using ThermoScript RT-PCR System (Invitrogen) using primers containing *Bsm*BI and *Bsa*I sites respectively, and cloned into pHW-2000 vector [57] according to the manufacturer's instructions. S203T, Q223R and H496N were introduced into the HA gene to generate pHW-2000-A/Tasmania/2004/2009 HA vector. L191I and R223Q were then introduced to generate *ferret-adapted* pHW-2000-A/Tasmania/2004/2009 HA vector. S388T was introduced into the NA gene to generate pHW-2000-A/Tasmania/2004/2009 NA vector. Further mutations and adaptations were introduced as indicated in the text. Mutations were introduced using the QuikChange II Site-Directed Mutagenesis kit (Strategene) with the QuikChange Primer Design Program.

### Pyrosequencing assay to quantify the proportion of the wildtype, N156K or K142N+N156K mutants

Two separate pyrosequencing assays were utilized. The first quantified the proportion of the N156 (wildtype) vs the N156K mutant (WT/N156K). The second assay quantified the proportion of K142 vs K142N mutant (N156K/K142N+N156K). A 350 bp region of the HA1 gene flanking amino acid positions 142 and 156 was amplified from plasmid mixtures and from cDNA from ferret peak day nasal wash samples using MyTaq HS Mix (Bioline) with biotinylated forward primer (5′Biotin-GCAATTGAGCTCAGTGTCATC) and reverse primer (5′TTCCGGCTTGAACTTCTTGC) (for the WT/N156K assay) or forward primer (5′GCAATTGAGCTCAGTGTCATC) and biotinylated reverse primer (5′Biotin-TTCCGGCTTGAACTTCTTGC) (for the N156K/K142N+N156K assay), according to the manufacturer's instructions. The pyrosequencing reaction was performed as previously described [58], using internal primers 5′GGATTTGCTGAGCTTTGGGT and 5′CTCATGCTGGAGCAAA, specific for the N156K and K142N mutations, respectively. The proportions of N156 and K156, or K142 and N142, HA in the sample, were estimated by calculating the ratio of the two peaks representing the N156 and K156, or K142 and N142 HA, using the AQ mode in the PyroMark ID 1.0 software. The N156K mutation generates a quadruplicate run of adenosines, which does not quantify absolutely by this technology [59]–[61], thus the detection limit was 5–10% for N156K. To correct for the sensitivity of the assay, all nasal wash samples were run with a set of plasmid mixtures containing various ratios. Mixtures of pGEMT plasmids with A/Tasmania/2004/2009-HA1 and A/Tasmania/2004/2009-HA1-N156K or A/Tasmania/2004/2009-HA1-N156K and A/Tasmania/2004/2009-HA1-K142N+ N156K inserts were prepared at different molar ratios (100∶0, 80∶20, 50∶50, 20∶80, 0∶100), at multiple concentrations (1, 0.1, 0.001 pg total). A standard curve was generated using the plasmid mixtures and the proportion of N156/K156 or K142/N142 in each nasal wash sample was determined by comparison to a standard curve. All samples were run in triplicate.

### Analysis of variants by cloning

The HA1 gene was amplified from cDNA generated from ferret nasal washes or egg-grown A/Tasmania/2004/2009 using the Velocity PCR kit (Bioline) with M13 tagged-HA1-specific primers (as above). Amplified product of the correct size was purified using the E-Gel CloneWell system (Invitrogen) and cloned using the Zero Blunt TOPO Cloning Kit for Sequencing (Invitrogen). DNA from 96 individual colonies with confirmed HA insert was sequenced for all samples except the initial inoculum for which 480 colonies were sequenced. HA1 was amplified using MyTaq HS Mix (Bioline) and A/Tasmania/2004/2009 HA1-specific primers ((F 5′ TAGTTCTGCTATATACATTTGCAACCG, R-5′GGATGTATATTCTGAAATGGGAGGC) then sequenced using the same primers. Poor quality sequences were excluded from the data set. As a control, the pGEMT-A/Tasmania/2004/2009 HA1 plasmid was transcribed using the Riboprobe In Vitro Transcription Systems (Promega), cDNA synthesized as above, then the HA1 gene amplified and cloned; 96 individual colonies were sequenced. The error rate in the amplification system, as determined by sequencing 96 clones from a single plasmid, was less than 0.001%.

### Reverse genetics rescue of influenza A viruses

Engineered viruses were generated as previously described [57], with infectious virus detected by hemagglutination and real time RT-PCR of culture supernatants. All viruses contained the HA and NA from ferret-adapted A/Tasmania/2004/2009 including specified mutations, whilst the remaining genes were from A(H1N1) A/Puerto Rico/8/1934 (pHW2000-A/Puerto Rico/8/1934 vectors, a kind gift from St Jude Children's Research Hospital, Memphis, TN, USA) or A(H1N1)pdm09 A/Perth/261/2009.

### Virus infection of cell cultures and analyses

Cells were seeded into T25 flasks or 24-well tissue culture plates and grown to confluence overnight. Monolayers were washed twice with Ca^2+^/Mg^2+^-free phosphate-buffered saline before incubation with 500 or 100 µl virus, respectively (nasal wash samples inoculated at 1/6 to 1/10 dilution; cell culture isolates inoculated neat; egg-grown virus inoculated at 1/100) at 37°C and 5% CO_2_ for 30 min, or other temperatures, as indicated. The inoculum was removed and replaced with medium (without fetal calf serum) supplemented with 4 µg/ml trypsin (Sigma) [52]. 2 mU/ml exogenous neuraminidase (from Clostridium perfringens (Sigma) or 5 nM oseltamivir carboxylate (the active form of the ethyl ester prodrug oseltamivir phosphate, kindly provided by Hoffmann-La Roche Ltd, Switzerland) was added to cultures as indicated in the text. Supernatant was collected daily and the presence of virus was assessed by hemagglutination using 1% turkey RBC and by real time RT-PCR assays for the influenza A matrix gene. Infection was also assessed by surface staining of infected cells and analysis by flow cytometry. Cells were stained with anti-influenza A HA monoclonal antibodies (mAbs) (A(H1N1)pdm09: mAb174 clone 10F5.1D7 and mAb175 clone 2G10.1C11; A(H3N2): mAb131 clone 4E4.1F10 and mAb 132 clone 1G6.1G7; kindly provided by CSL Limited), ferret antisera, followed by anti-ferret Ig-FITC (Rockland Immunochemicals Inc., USA) or anti-influenza A matrix mAb (clone GA2B, AbD Serotec, UK), followed by anti-mouse Ig-FITC (SantaCruz Biotechnology and KPL). Staining was performed in the presence of 1 µM oseltamivir carboxylate. Samples were run on a FC500 Analyzer (Beckman Coulter) or FACSCanto II (BD Biosciences) with data analysis using FlowJo 7.5.5 software.

### Flow cytometry-based glycan binding assay

The binding specificity of influenza HA was determined using an assay previously described [41]. Briefly, MDCK-SIAT1 cell monolayers, in 24-well plates, were infected with the same copy number of influenza virus from different ferret nasal washes, or reverse genetics viruses. After 48 h, cells were harvested, washed three times with PBS and stained with biotinylated multivalent glycans (5 µg/ml) and mouse anti-influenza A matrix mAb in PBS, with 1 µM oseltamivir carboxylate for 2 h at 4°C. Glycans, Neu5Acα2-6Galβ1,4GlcNAc-PAA-biotin (6′ sialyllactosamine; 6′SLN), Neu5Acα2-3Galβ 1,4Glc-PAA-biotin (3′ sialyllactose; 3′SL) and Neu5Acα2-6Galβ 1,4Glc-PAA-biotin (6′ sialyllactose; 6′SL), were purchased from GlycoTech (MD, USA). To detect biotinylated glycan binding, cells were incubated with streptavidin-RPE (Vector Laboratories, USA) and mouse mAb was detected with anti-mouse Ig-FITC in PBS with 1 µM oseltamivir carboxylate, for 1 h at 4°C. Samples were analysed by flow cytometry. Cell culture supernatant was also collected upon cell harvest and assessed by real time RT-PCR and hemagglutination. HA and NA genes were sequenced to ensure no adaptations had occurred.

### Hemagglutination Inhibition (HI) assay

Reactivity of serum samples was measured using HI assays [62] as described elsewhere [63]. Titres were expressed as the reciprocal of the highest dilution of serum for which hemagglutination was prevented. GMT was calculated with undetectable titres expressed as ‘5’. Seroconversion was defined as titre ≥40.

### Statistics

Viral growth kinetics were assessed using real time RT-PCR data. Animals artificially infected by intranasal inoculation were excluded from the analysis. The virus growth rate was calculated as the difference between viral copy number between the peak day and the first day, divided by the number of days to reach peak viral copy number. Animals for which viral load peaked on day 1 were excluded. The serial interval between transmission was defined as the number of days between virus detection in the infecting and infected ferret. Only transmission events where both animals were deemed infected using the same method of detection were included. Transmission events involving artificially inoculated animals or interruptions (see results) were excluded. Data were analysed using one-way ANOVA, and Bonferonni correction to adjust for multiple comparisons, using STATA. Ferret antisera binding to virus-infected cells was analyzed by comparing the relative proportion from wildtype and N156K-infected cells for each antiserum using Spearman's rank correlation co-efficient. Statistically significant results are indicated with * p<0.05, ** p<0.01 and *** p<0.005.

### Analysis of frequency of mutations in human clinical specimens on GISAID and Genbank

All A(H1N1)pdm09 HA sequences from viruses isolated from a human host since 2009 were downloaded from GISAID and GenBank, and filtered to remove strains occurring multiple times. The resulting 16740 sequences were aligned using MAFFT [64]. To determine whether a mutation preferentially occurred in a culture type, sequences with passage history were classified into four categories based on the source from which they were extracted and sequenced: (i) embryonated hen's egg, (ii) MDCK cells, (iii) MDCK-SIAT1 cells and (iv) original clinical sample. The odds ratios were calculated from the probabilities of occurrence of the mutant and the wildtype in each of the source cell types. The degree of association of the mutants to the source cell types were classified into 5 categories: (i) moderate association (1.5–3), (ii) strong association (>3.0), (iii) moderate negative association (0.33–0.66), (iv) strong negative association (<0.33) and (v) no association (0.66–1.5) [65].

### 3-D modeling

Simulated annealing energy minimizations through short molecular dynamics (MD) simulations using the AMBER03 force field in YASARA were done for wildtype and mutant HAs in complex with either α-2,3 or α-2,6 ligand (PDBs: 3ubj chains C and D or 3ube chains A and B, respectively [66]. For structural correspondence, only the sialic acid and the first bound galactose of the ligands were kept. The ligands were parameterized automatically by YASARA [67]. YASARA has been previously shown to provide excellent results in the CASP competition for homology model refinements [68]. The used minimization protocol comprises the following steps: in short, to remove bumps and correct the covalent geometry, the structure was energy-minimized with the AMBER03 force field [69], using a 7.86431 Angstroem force cutoff and the Particle Mesh Ewald algorithm [70] to treat long-range electrostatic interactions. After removal of conformational stress by a short steepest descent minimization, the procedure continued by simulated annealing (time step 2 fs, atom velocities scaled down by 0.9 every 10th step) until convergence was reached, i.e. the energy improved by less than 0.05 kJ/mol per atom during 200 steps. Counterions are implicitly considered by setting the net charges to 0. Solvation is also considered implicitly by a term proportional to the accessible surface area. 1.2 kJ/mol was used as the estimate for the entropic cost of exposing one square Angstroem to the solvent. To further reduce the computational complexity and potential unrelated simulation bias, atoms further than 8 Angstroem from either the ligand or the respective mutated residues were fixed (not allowed to move). This resulted in a system of both flexible ligand and flexible receptor binding pocket. The relative binding energies were estimated through a function considering standard potential energy terms plus the above mentioned implicit solvation. Three steps of sequential forward and backward mutations for each mutation set were employed and averaged over the visited energy minima. Finally, for each analyzed single or pair mutation(s) we subtracted the predicted energy for binding the α-2,6 receptor from the predicted energy for binding the α-2,3 receptor. Higher values of the measure therefore indicate α-2,6 preference while lower values would point to relative α-2,3 preference. In order to compare the predicted measure with the experimental glycan binding data (see above), we derived a corresponding relative preference value by subtracting the mean fluorescence intensity of glycan expression for 3′SL from the average for 6′SL and 6′SLN for the respective mutation(s). The subtraction order of experimental receptor data was inversed to obtain a direct linear correlation with the computational measure.

## Supporting Information

Figure S1 **Time course of influenza transmission through B passage lines.** A separate D0 and D1 ferret established the passage line B of the naïve experimental group. Similarly, a separate D0 and D1 ferret established passage line B of the MIV experimental group. The same D0 ferret infected two different D1 ferrets to establish the passage line B of the PBS+IFA and MIV+IFA experimental groups. Nasal washes were collected daily from ferrets and virus load measured by real time RT-PCR. During the experiment, both the rapid test result (PBS+IFA, MIV+IFA day 0–26) and the raw Ct value (naïve, MIV, MIV+IFA day 27 onwards) was used as a marker of infection and transmission. The data points whereby transmission of virus to recipient ferrets were deemed to have occurred are identified as red symbols. Direct intranasal inoculation (arrow).
(TIF)

Figure S2 **Time course of N156K mutant influenza virus transmission through naïve ferrets.** R0–R7 ferrets from lines **A** and **B** are identified. Nasal washes were collected daily from ferrets and virus load measured by real time RT-PCR assay. During the experiment, the raw Ct value was used as a marker of infection and transmission. The data points whereby transmission of virus to recipient ferrets were deemed to have occurred are identified as red symbols. Direct intranasal inoculation (arrow).
(TIF)

Figure S3 **3-D modeling of structure and interactions around HA position 156.** **(A)** Linear correlation of experimentally measured and computationally predicted relative α-2,6- to α-2,3-linked receptor preference, R^2^ = 0.72 **(B)** Position 156 (red) on the HA head domain (gray) is at the crossing of 3 previously crystallized antibody binding interfaces (cyan-antibody to 1918 A(H1N1) PDB:3lzf [48]; yellow-antibody to A(H3N2) PDB:2vir [47]; green-antibody to A(H3N2) PDB:1ken [46]. **(C–D)** 3-D modeling of HA containing G155E+N156K. **(C)** Electrostatic surface potential in the HA head domain, calculated with the Particle Mesh Ewald method implemented in YASARA. Blue indicates positive and red indicates negative charge potential. A host receptor analogue is shown in magenta. **(D)** Structural modeling of single and pair mutations in HA with bound α-2,6- or α-2,3-linked host receptor ligands. Comparison of N156 wildtype (green HA/yellow ligand) and N156K (cyan HA/red ligand) with double mutant G155E+N156K (purple HA/gray ligand).
(TIF)

Table S1 **HA1 genetic variation within individual nasal wash samples and virus inoculum by cloning analysis.** Variation compared to the original egg inoculum consensus sequence and number of times mutation detected ^(#)^ is indicated. Bold mutations were detected in ≥4% of colonies sequenced.
(DOCX)
