# Supplementary figures and images for: Antigenic Drift of the Pandemic 2009 A(H1N1) Influenza Virus in a Ferret Model
Source: PLoS Pathog. 2013 May 9;9(5):e1003354. doi: 10.1371/journal.ppat.1003354 (PMC3649996; doi:10.1371/journal.ppat.1003354)

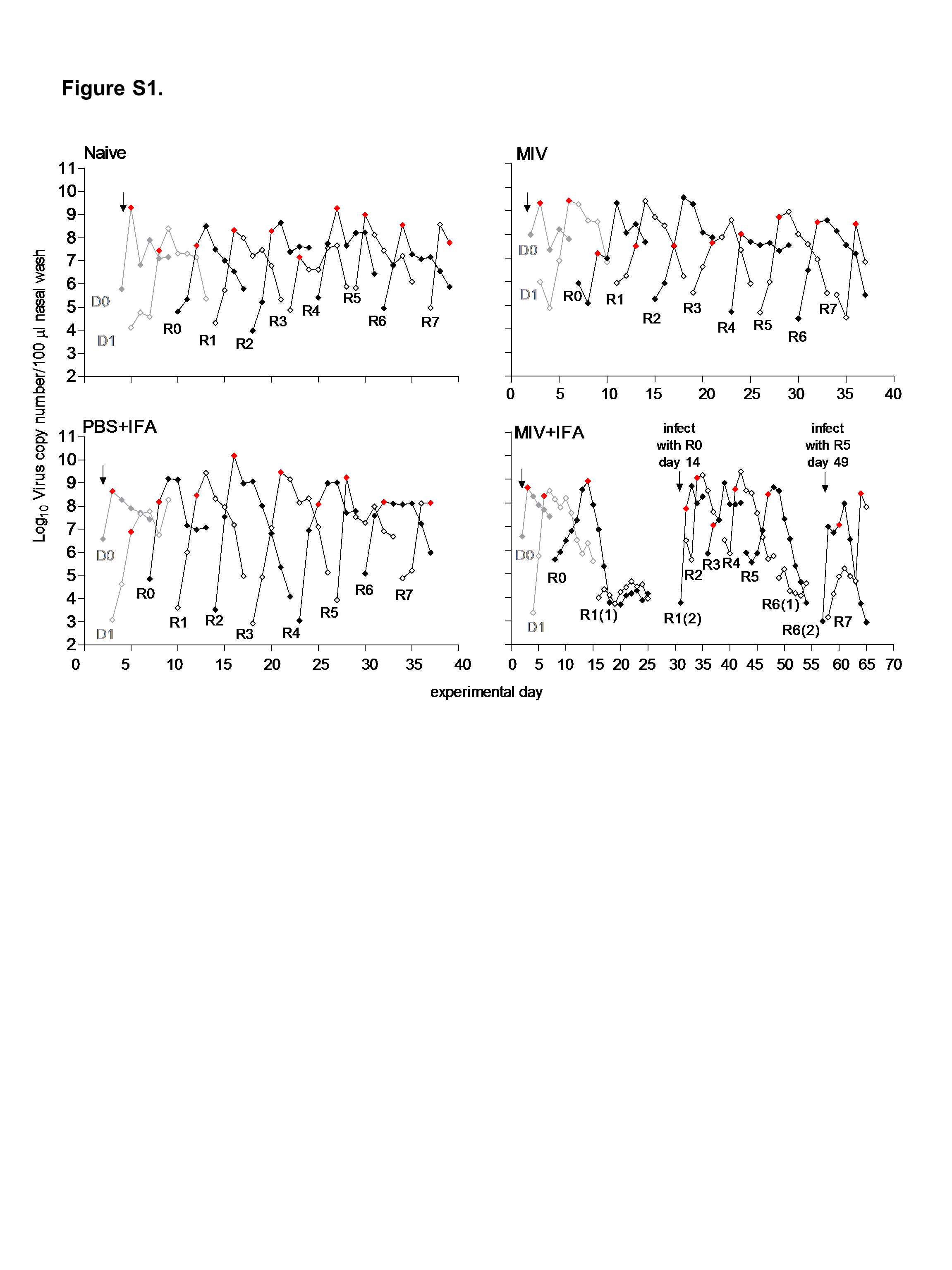

Supplement: Figure S1 — Time course of influenza transmission through B passage lines. A separate D0 and D1 ferret established the passage line B of the naïve experimental group. Similarly, a separate D0 and D1 ferret established passage line B of the MIV experimental group. The same D0 ferret infected two different D1 ferrets to establish the passage line B of the PBS+IFA and MIV+IFA experimental groups. Nasal washes were collected daily from ferrets and virus load measured by real time RT-PCR. During the experiment, both the rapid test result (PBS+IFA, MIV+IFA day 0–26) and the raw Ct value (naïve, MIV, MIV+IFA day 27 onwards) was used as a marker of infection and transmission. The data points whereby transmission of virus to recipient ferrets were deemed to have occurred are identified as red symbols. Direct intranasal inoculation (arrow). (TIF) [file ppat.1003354.s001.tif]

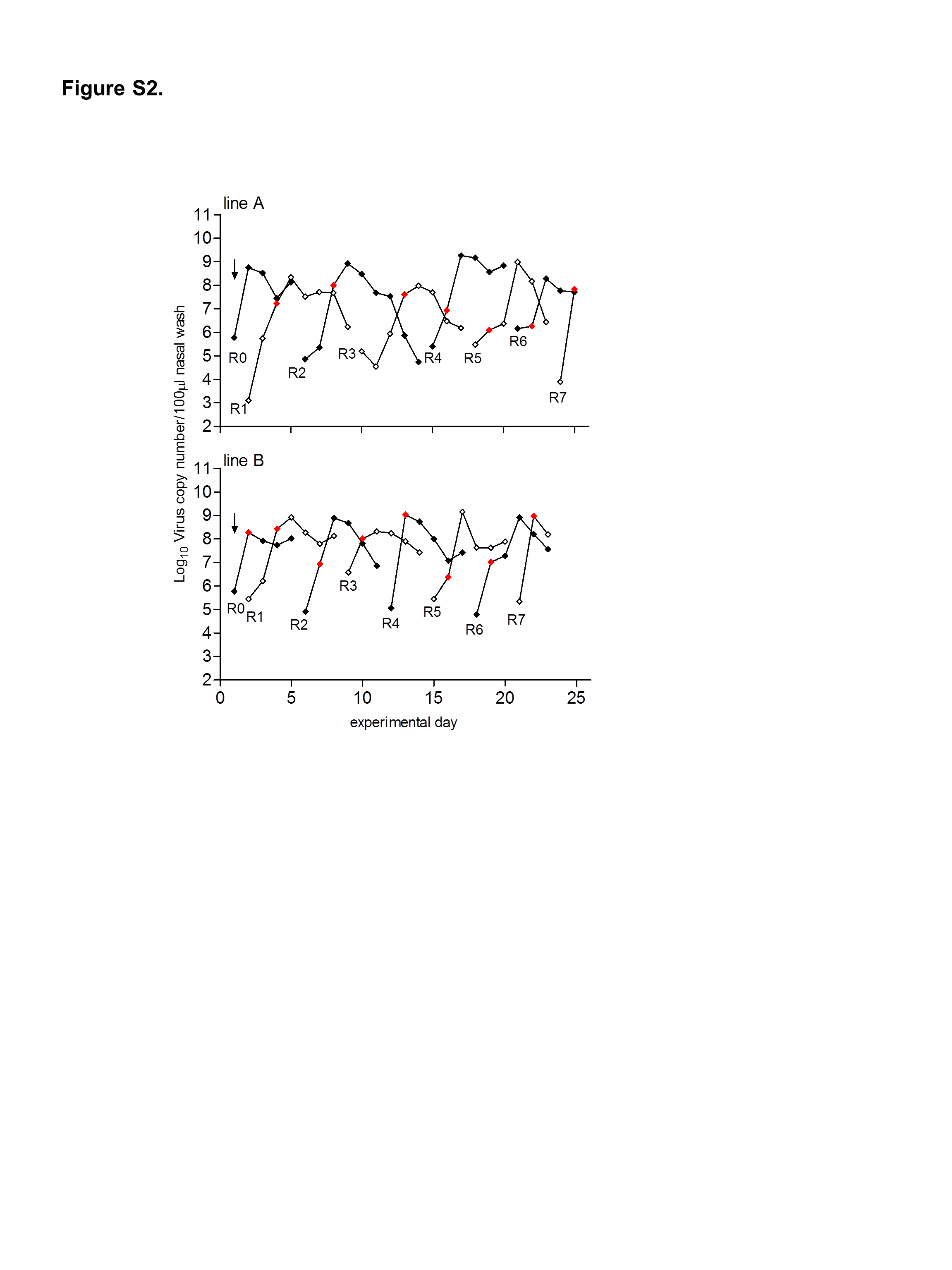

Supplement: Figure S2 — Time course of N156K mutant influenza virus transmission through naïve ferrets. R0–R7 ferrets from lines A and B are identified. Nasal washes were collected daily from ferrets and virus load measured by real time RT-PCR assay. During the experiment, the raw Ct value was used as a marker of infection and transmission. The data points whereby transmission of virus to recipient ferrets were deemed to have occurred are identified as red symbols. Direct intranasal inoculation (arrow). (TIF) [file ppat.1003354.s002.tif]

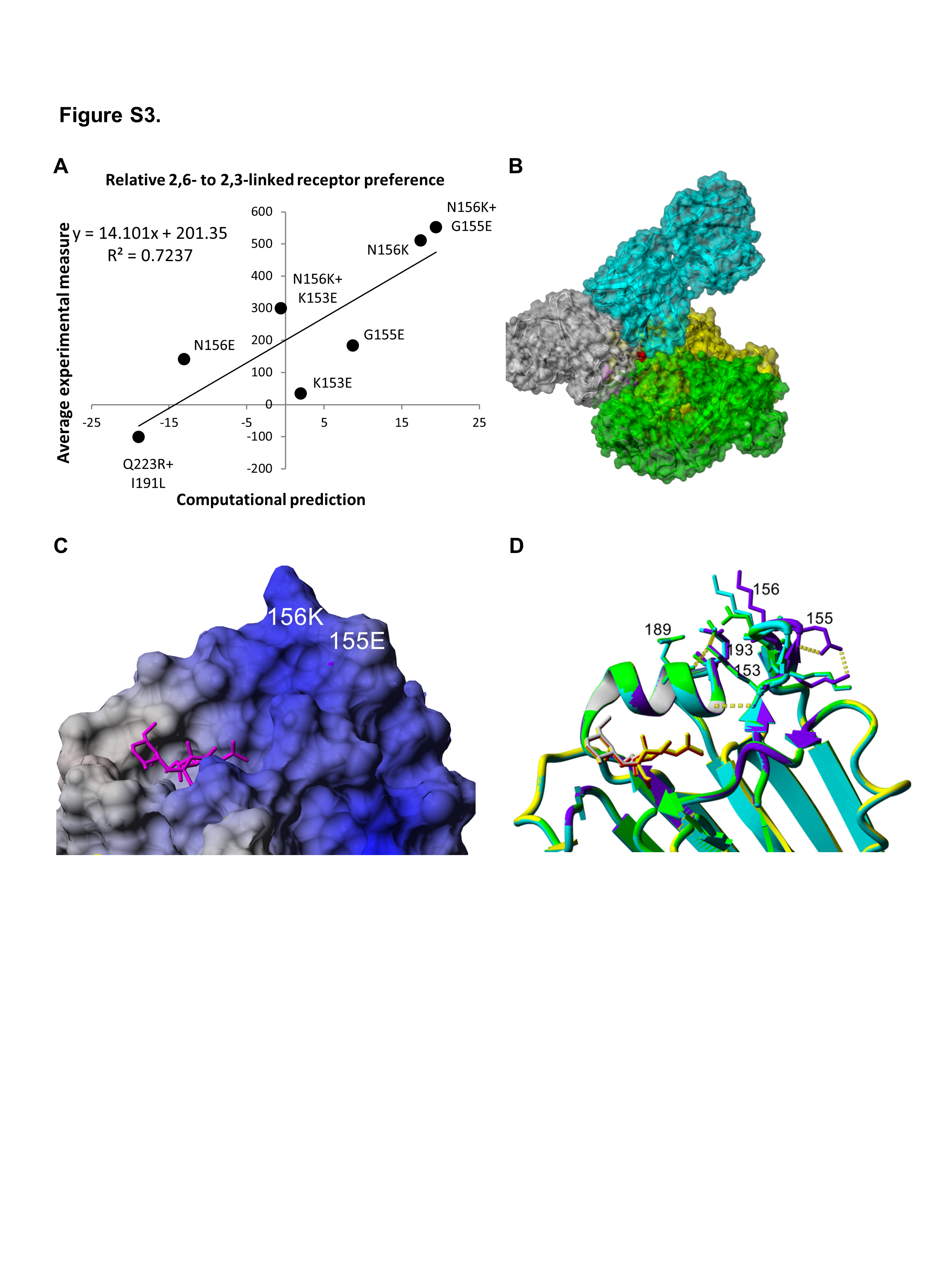

Supplement: Figure S3 — 3-D modeling of structure and interactions around HA position 156. (A) Linear correlation of experimentally measured and computationally predicted relative α-2,6- to α-2,3-linked receptor preference, R2 = 0.72 (B) Position 156 (red) on the HA head domain (gray) is at the crossing of 3 previously crystallized antibody binding interfaces (cyan-antibody to 1918 A(H1N1) PDB:3lzf [48]; yellow-antibody to A(H3N2) PDB:2vir [47]; green-antibody to A(H3N2) PDB:1ken [46]. (C–D) 3-D modeling of HA containing G155E+N156K. (C) Electrostatic surface potential in the HA head domain, calculated with the Particle Mesh Ewald method implemented in YASARA. Blue indicates positive and red indicates negative charge potential. A host receptor analogue is shown in magenta. (D) Structural modeling of single and pair mutations in HA with bound α-2,6- or α-2,3-linked host receptor ligands. Comparison of N156 wildtype (green HA/yellow ligand) and N156K (cyan HA/red ligand) with double mutant G155E+N156K (purple HA/gray ligand). (TIF) [file ppat.1003354.s003.tif]
