# Supplementary material for: Antigenic Drift of the Pandemic 2009 A(H1N1) Influenza Virus in a Ferret Model
Source: PLoS Pathog. 2013 May 9;9(5):e1003354. doi: 10.1371/journal.ppat.1003354 (PMC3649996; doi:10.1371/journal.ppat.1003354)
Supplement: Table S1 — HA1 genetic variation within individual nasal wash samples and virus inoculum by cloning analysis. Variation compared to the original egg inoculum consensus sequence and number of times mutation detected (#) is indicated. Bold mutations were detected in ≥4% of colonies sequenced. (DOCX) [file ppat.1003354.s004.docx]

***Table S1. HA1 genetic variation within individual nasal wash samples and virus inoculum by cloning analysis.***

|  |  |  |  | **Amino acid mutations** | | | |
| --- | --- | --- | --- | --- | --- | --- | --- |
| **Sample** | **No. colonies sequenced** | **No. mutations** | **No. synonymous mutations** | **N156** | **Also in antigenic sites, Sa, Sb, Ca, Cb [**[**6**](#_ENREF_6)**]** | **Implicated in receptor specificity or antibody binding [**[**30**](#_ENREF_30)**,**[**31**](#_ENREF_31)**]** | **No described function** |
| pGEMT-A/Tasmania/2004/2009 HA1 | 96 | 2^c^ | 0 | - | **L191I^96a^** | **R223Q^96a^** | 0 |
| A/Tasmania/2004/2009 inoculum | 478^b^ | 34 | 9 | - | A83T^1^, K142E^1^, K153R^1^, K169N^1^, **L191I^57a^**, N194D^1^, A195V^1^ | **R223Q^53a^** | N31D^1^, **N38S^130^**, G46R^1^, H51Q^1^, I61V^1^, P92T^1^, F95Y^1^, E99G^1^, K119Q^2^, S128P^1^, A135G^1^, Y165_STOP_^1^, L176P^1^, P271S^1^, Y229_STOP_^1^, D269N^2^, D274N^1^ |
| Naïve A R7 | 94 | 8 | 0 | - | **L191I^94a^**, R205G^3^ | **G131S^94^, R223Q^94a^** | F95S^1^, D196G^3^, T245N^1^, H273P^1^ |
| MIV A R7 | 96 | 6 | 1 | - | **L191I^96a^** | **D187V^96^, R223Q^96a^** | G202W^1^, C275Y^1^ |
| PBS+IFA A R7 | 95 | 8 | 0 | - | S71T^1^, **L191I^95a^** | **R223Q^95a^** | Y7C^1^, E81G^1^, R113M^1^, S122_STOP_^1^, H180R^1^ |
| MIV+IFA A R4 | 95 | 15 | 4 | **N156K^55^** | P137L^1^, G155R^1^, **L191I^96a^** | **R223Q^95a^** | **D14E^96^**, C90_STOP_^1^ , N129D^1^, P182Q^1^ , N228K^1^, R252G^1^ |
| MIV+IFA A R7 | 96 | 10 | 2 | **N156K^96^** | G155R^1^, **L191I^96a^**, E235K^1^ | **R223Q^96a^** | (D14E)^d^, A134E^3^, C90F^1^, S143G^1^ |
| MIV+IFA B R1 | 93 | 9 | 4 | **N156K^52^** | **L191I^93a^** | **R223Q^93a^** | S121N^1^, G178D^1^ |
| MIV+IFA B R7 | 96 | 9 | 3 | **N156K^96^** | N125S^1^, **L191I^96a^** | **R223Q^96a^** | E99D^1^, S207G^1^ |
| N156K Naïve A R7 | 96 | 10 | 3 | **N156K^96^** | K142T^1^, **L191I^96a^** | **R223Q^96a^** | **D14E^96^**, L105F^1^, R113Q^1^ |
| N156K Naïve B R7 | 95 | 7 | 1 | **N156K^95^** | K142N^95^, **L191I^96a^** | **R223Q^96a^** | S12L^1^, S106N^1^ |

Variation compared to the original egg inoculum consensus sequence and number of times mutation detected ^(#)^ is indicated. Bold mutations were detected in ≥ 4% of colonies sequenced.

^a^site implicated in receptor binding/host specificity

^b^Five separate ligations performed (95-96 clones sequenced from each ligation). Mutations which were detected more than 5 times were detected in colonies from all five ligations in similar proportions i.e.ligation 1=g291a^5^, N38S^27^, L191I^9^, R222Q^12^, ligation 2= g291a^6^,N38S^28^, L191I^11^, R222Q^8^, ligation 3= g291a^6^,N38S^30^, L191I^5^, R222Q^5^, ligation 4= g291a^1^,N38S^24^, L191I^16^, R222Q^13^, ligation 5= g291a^2^,N38S^21^, L191I^16^, R222Q^15^

^c^L191I and R223Q are present in the ferret-like pGEMT-A/Tasmania/2004/2009 HA1 vector

^d^() mutation detected by Sanger sequencing, but outside limits of sequence data
